# Clinical features and HLA typing of immune checkpoint inhibitor-associated myasthenia gravis, myocarditis and myositis

**DOI:** 10.3389/fonc.2025.1646231

**Published:** 2025-11-21

**Authors:** Xiaoting Lin, Peng Liu, Min Jin, Guoyan Qi

**Affiliations:** 1 Treatment Center of Myasthenia Gravis, People’s Hospital of Shijiazhuang, Shijiazhuang, Hebei, China; 2 Hebei Provincial Key Laboratory of Myasthenia Gravis, Shijiazhuang, Hebei, China; 3 Hebei Provincial Clinical Research Center for Myasthenia Gravis, Shijiazhuang, Hebei, China; 4 School of Basic Medicine, North China University of Science and Technology, Tangshan, Hebei, China

**Keywords:** immune checkpoint inhibitors, immune-related adverse events, HLA, myasthenia gravis, myocarditis, myositis

## Abstract

**Background:**

Immune checkpoint inhibitors (ICI) have significantly improved the therapeutic outcomes for various malignant tumors, but they may also trigger severe immune-related adverse events (irAEs), such as myositis, myocarditis and myasthenia gravis (MG). The mechanisms underlying these adverse reactions remain unclear and may be related to an individual’s genetic background. Human Leucocyte Antigen (HLA) genes play a crucial role in immune regulation, and their polymorphism is closely related to the occurrence of various autoimmune diseases.

**Method:**

To clarify whether irAEs is associated with specific HLA typing, we analyzed 23 patients diagnosed with myositis, myocarditis and/or MG after receiving ICI therapy, and conducted HLA high-resolution typing detection using the PCR-SBT method.

**Results:**

Among the 14 patients with MG combined with myositis and myocarditis, the frequencies ofHLA-DQB1*03:03 and HLA-C*01:02 loci were higher than those in the normal population. In 9 patients with myositis and myocarditis, the frequency of HLA-B*52:01 locus was higher than that in the normal population.

**Conclusion:**

Our research findings reveal that patients carrying autoimmune susceptibility genes have a higher risk of developing irAEs when treated with ICI, specific HLA alleles may be associated with the risk of ICI-related MG/myositis/myocarditis, but further large-scale, multi-center validation is needed.

## 1 Introduction

Immune checkpoint inhibitors (ICI) have been widely used in the treatment of various advanced cancers at present, significantly prolonging the survival time of cancer patients (1). Programmed cell death-1/programmed cell death ligand-1 (PD-1/PD-L1) and cytotoxic T-lymphocyte-associated antigen 4 (CTLA-4) blockers and other ICI have been listed as standard treatment options for multiple types of cancer (2–4). ICI exerts anti-tumor effects by relieving immune suppression in the body and enhancing immune function (5). ICIs exert anti-tumor effects by relieving immune suppression and enhancing immune function. Despite their remarkable anti-tumor efficacy, ICIs may trigger extensive, life-threatening inflammatory and immune-related adverse events (irAEs), which are due to their induction of off-target inflammatory and autoimmune responses (6).

Neurological adverse events related to ICI treatment are relatively rare. Myasthenia gravis (MG), a disease caused by dysfunction of neuromuscular junction transmission, is also a type of irAEs in the nervous system. ICI treatment-induced MG is known as immune-related MG (irMG), with an incidence rate of approximately 0.12% to 0.2% (7). The incidence rate in Asia may be higher than that in Western countries (8). irMG can either be an exacerbation or recurrence of pre-existing MG (with a clear history of MG before ICI treatment), or it can be a newly developed MG after ICI treatment (with no previous history of MG but developing after ICI treatment). irMG alone is relatively rare, more often irMG is combined with myositis and/or myocarditis and the latter more often progresses rapidly to a MG crisis requiring intensive monitoring and mechanical ventilatory support (9). ICI-induced myocarditis is a rare and serious adverse event, with an incidence of less than 1% of irAEs reported in clinical trials and institutional series (10), with some studies reporting up to 30% - 40% and 10% incidence of concomitant myositis and MG, respectively, in patients with immune-associated myocarditis (11). Immune-related myotoxicity (immune-related myositis and/or myocarditis) has the highest lethality of all irAEs, approximately >20% (12), and up to 60% in cases of concomitant myocarditis (13). The low prevalence of irMG combined with myositis and/or myocarditis and the limited number of described cases limit our ability to describe the clinical features and outcomes of this disease and to optimize its diagnosis and management.

Accumulating evidence suggests that patient response to ICI therapy is not universal, and the group of patients with a dual diagnosis (autoimmune disease and cancer) who require the use of ICI is quite challenging (14). It is critical to identify the determinants of response to ICI therapy, adverse effects, which can be used as prognostic and predictive markers (15). The development of irAEs is dependent on a number of complex factors associated with overactivation of the immune system, expression of ectopic antigens, antibody-dependent responses, increased cytokines, and genetic predisposition (16). Genetic predispositions include polymorphisms in immune-related genes and therapeutic target polymorphisms (17), deficiencies in the expression of Human Leukocyte Antigen (HLA) molecules, and impaired antigen processing and presentation (18). HLA gene polymorphisms are closely associated with the development of various autoimmune diseases, and HLA molecules play a central role in antigen presentation and the initiation of specific immune responses (19). Some studies have shown the importance of HLA variants in ICI treatment response (20, 21). There are few reports on the HLA genotyping of ICI-associated myositis, myocarditis, and MG. By analyzing the correlation between HLA genotyping and the occurrence of ICI-associated myositis, myocarditis, and MG, it can provide a basis for the early identification of high-risk patients and the development of individualized treatment plans, thus reducing the incidence of irAEs and improving the safety of patient treatment and the quality of survival.

## 2 Materials and methods

### 2.1 Patient information

Data were collected from patients who attended the Myasthenia Gravis Centre of Shijiazhuang People’s Hospital in 2020-2024, with the following inclusion criteria (1): any type of immune checkpoint inhibitor used before the onset of myositis, myocarditis, or MG (2); confirmed or suspected diagnosis of myositis, myocarditis, or new-onset MG or worsening of symptoms of MG in well-controlled conditions; and (3) patients with complete clinical data.

The diagnosis of MG is made on the basis of typical clinical features of MG (fluctuating muscle weakness) and any one of the following three points, including a positive neostigmine test, electrophysiological features, and serum antibodies such as AChR-Ab.

The diagnosis of myositis was defined as follow: CK elevation exceeding the upper reference limit (CK threshold: The reference range of the Laboratory of Shijiazhuang People’s Hospital was adopted, with 50–310 U/L for males and 40–200 U/L for females). Compatible clinical symptoms (e.g., proximal muscle weakness, myalgia). Supportive evidence from at least one of the following: Electromyography (EMG) showing myopathic changes: Fibrillation potentials/positive sharp waves were observed at rest. The motor unit action potentials exhibited characteristics of myopathic changes, including short duration (<10 ms), low amplitude (<500 μV), and an increased proportion of polyphasic waves (>20%); Magnetic Resonance Imaging (MRI) showing features of muscle inflammation: High signal intensity in the myofascia or intramuscular regions was observed on T2-weighted imaging with fat saturation (T2WI-FS); Muscle biopsy confirming inflammatory infiltrate: Lymphocytic infiltration in the muscle interstitium. To ensure that the increase in CK is due to the inflammatory response induced by ICI rather than other causes, we have established a multi-dimensional screening process: Temporal correlation analysis: Elevated CK in all included patients occurred after ICI treatment, and abnormal CK before treatment was excluded. Exclusion of other causes: Other common causes of CK elevation need to be excluded, such as physical, chemical, and biological factors, as well as systemic or other systemic diseases: Muscle injury: No recent history of intense exercise or trauma. Drug-induced muscle damage (such as statins). Infectious myositis (such as viral, bacterial infections). Endocrine myopathies (such as hypothyroidism).

In myocarditis, endomyocardial biopsy (EMB) is the diagnostic gold standard, but the invasive nature and procedural risks limit widespread use. Accordingly, diagnosis is based on clinical and biomarker assessment, aided with echocardiography and CMR. The diagnostic criteria for ICI associated myocarditis are as follows: Myocarditis occurs after ICI treatment. Excluding non-ICI treatment-related myocarditis (such as viral myocarditis, autoimmune myocarditis) and other cardiac complications (such as myocardial infarction). Clinical presentation (e.g., chest pain, dyspnea). Cardiac biomarker elevation (e.g., troponin I. The reference range of the Laboratory of Shijiazhuang People’s Hospital was adopted, with 0–0.0198 ng/mL for males and 0–0.0116 ng/mL for females). Objective evidence of cardiac dysfunction or inflammation from at least one of the following: Echocardiographic findings (new reduction in LVEF, etc). Cardiac MRI findings consistent with myocarditis, Myocardial edema on T2WI (high signal intensity); Late gadolinium enhancement (LGE) showing non-ischemic myocardial necrosis/fibrosis. Electrocardiographic (ECG) changes (new arrhythmia, conduction delay, ST/T-wave changes, etc). Coronary angiography (the coronary arteries of patients with myocarditis usually show no significant stenosis, which can be used to distinguish it from myocardial infarction).

The study was approved by the Ethics Committee of Shijiazhuang People’s Hospital. Written informed consent was obtained from all participants.

### 2.2 Data collection

We extracted variables on patient demographics and baseline characteristics, including age, sex, ICI type, ICI indication, time from onset to first ICI injection and severity of irAEs, clinical manifestations of MG (ptosis, diplopia, dyspnea, limb weakness, dysphagia, etc.), laboratory test values (anti-acetylcholine receptor, creatine phosphokinase), treatment options, prognosis.

The clinical severity of irMG was assessed using the Myasthenia Gravis Foundation of America (MGFA) grading system. The MGFA grading: grade I: ocular muscle weakness only; grade II: mild non-ocular muscle weakness; grades III and IV were defined as moderate and severe non-ocular muscle weakness, respectively; and grade V was defined as the need for intubation with or without mechanical ventilation except for intubation used during routine postoperative management. Based on whether the patient has a history of MG in the past, irMG is classified into new-onset MG and past MG. The severity of irAEs is graded according to the common terminology criteria for adverse events (CTCAE), which classify adverse reactions into five grades, from mild to severe: Grade 1 (mild): Symptoms are mild and may not require treatment, but close monitoring is necessary. Grade 2 (moderate): Symptoms are significant and require medication, possibly affecting daily life. Grade 3 (severe): Symptoms are severe, self-care is limited, and hospitalization is required. Grade 4 (life-threatening): Symptoms are life-threatening and require urgent intervention. Grade 5 (death): Death related to toxicity. The outcome of irAEs after treatment is defined as complete remission, improvement, no change or deterioration/death.

### 2.3 HLA genotyping assay

3 ml of venous blood samples were collected from the patients using the vacuum tube sampling method without additives. The HLA high-resolution typing test was conducted by PCR-SBT method and the typing was referred to the IMGT/HLA database. A total of 12 alleles were tested: *HLA-A*, *B*, *C*, *DPA1*, *DPB1*, *DQA1*, *DQB1*, *DRB1*, *DRB3*, *DRB4*, *DRB5*, and *G*.

### 2.4 Statistical analysis

The statistical analyses were conducted using SPSS 28.0 software. We report allele frequencies (number of alleles/2n), not carrier frequencies (number of patients carrying the allele). Comparisons of HLA allele frequencies between the patient group and the control group (the common and confirmed HLA allele table of the Chinese population (CWD2.4) published by the China Marrow Donor Program Management Center and the large sample HLA literature reports of the Han Chinese population in China) were tested for significance by using Fisher’s exact test. The effect size was expressed as an odds ratio (OR) with a 95% confidence interval (CI), *P* value <0.05 was considered statistically significant. Continuous variables were expressed as median and range, and categorical variables were expressed as frequency and percentage.

## 3 Results

### 3.1 Basic information

From 2020 to 2024, a total of 19,500 patients were treated at the Myasthenia Gravis Center of Shijiazhuang People’s Hospital. Among them, 25 patients met the diagnostic criteria for ICI-related myositis, myocarditis, and MG. Two patients were excluded due to incomplete clinical data, leaving a final cohort of 23 patients. All were of Han ethnicity, with 14 males (60.9%) and a median age of 64 (33–86) years. The basic characteristics of the patients are summarized in **Table 1**.

**Table 1:** Baseline characteristics of patients with irMG ± myositis and myocarditis (n = 23).

Baseline characteristics	Classification	n/median	(%)/ Interquartile range
Age	year	64	(33-86)
Gender	Male	14	60.9%
	Female	9	39.1%
Type of tumor	Thymoma	9	39.1%
	Esophagus cancer	3	13.0%
	liver cancer	3	13.0%
	Pulmonary adenocarcinoma	2	8.7%
	Gastric cancer	2	8.7%
	small cell lung cancer	1	4.3%
	Adenocarcinoma of the gastroesophageal junction	1	4.3%
	Colon cancer	1	4.3%
	Cervical cancer	1	4.3%
	Metastatic adenocarcinoma of the abdominal cavity	1	4.3%
ICI treatment regimen			
Anti-PDL1	Durvalumab	1	4.3%
Anti-PD1	Sintilimab	8	34.8%
	Camrelizumab	7	30.4%
	Tislelizumab	2	8.7%
	Toripalimab	1	4.3%
	Pembrolizumab	1	4.3%
	Paprimab	1	4.3%
	Sulriolimab	1	4.3%
	• Other	1	4.3%
Types of irAEs	irMG combined with myositis and myocarditis	14	60.9%
	Myositis and myocarditis	9	39.1%
Disease process			
The onset time after ICI exposure	days	15	(5-60)
Number of ICI treatment cycles	cycle number	1	(1-3)
irAEs classification	Level 1-2	0	0.0%
	Level 3	9	39.1%
	Level 4	13	56.5%
	Level 5	1	4.3%
Poor outcomes	Tracheotomy, tracheal intubation or death	6	26.1%
	non-invasive ventilator	5	21.7%
clinical symptoms	extraocular muscle involvement	20	87.0%
	neck and/or limb muscle weakness	15	65.2%
	Dysarthria or dysphagia	10	43.5%
	dyspnea	11	47.8%
laboratory test values	AChR-Ab	14	60.9%
	MuSK-Ab	0	0.0%
	Titin-Ab	4	17.4%
	RyR-Ab	1	4.3%
	Antinuclear-Ab	11	47.8%
	Peak creatine kinase(U/L)	2040	540-26285
treatment regimen	methylprednisolone	19	82.6%
	immunosuppressor	10	43.5%
	human immunoglobulin	11	47.8%
	chemotherapy	4	17.4%
	plasmapheresis	4	17.4%
	Efgartigimod	2	8.7%
	Temporary cardiac pacemaker	4	17.4%
prognosis	complete remission	13	56.5%
	improve	7	30.4%
	unchange	2	8.7%
	Deterioration and/or death	1	4.3%

22 patients received anti-PD-1 treatment and 1 received anti-PD-L1 treatment. The most common primary tumors treated with ICI were thymoma (n=9, 39.1%), followed by esophageal cancer and liver cancer (n=3 each, 13.0%), lung adenocarcinoma and gastric cancer (n=2 each, 8.7%), small cell lung cancer, gastroesophageal junction cancer, colon cancer, cervical cancer, and metastatic adenocarcinoma of the abdominal cavity (n=1 each, 4.3%). One patient with thymoma also had esophageal cancer. The pathological types and irMG types of the 9 thymoma patients are shown in **Table 2**. The most common type was B3 thymoma (n=3, 33.3%), and one patient with type A thymoma did not have irMG. 9 patients (39.1%) were diagnosed with myositis combined with myocarditis, and 14 patients (60.9%) were diagnosed with irMG combined with myositis and myocarditis. Among them, 5 patients had a previous history of MG, and 9 were new-onset MG. The MGFA classification and other details of the 14 irMG patients are shown in **Table 3**.

**Table 2:** Pathology and irAEs types of thymoma patients (n = 9).

Pathology	A	1	11.1%
	AB	1	11.1%
	B1	2	22.2%
	B2	2	22.2%
	B3	3	33.3%
irAEs types	Pre-existing MG	4	44.4%
	Newly developed MG	4	44.4%
	Without MG (myocarditis, myositis)	1	11.1%

**Table 3:** Clinical characteristics of irMG patients (n = 14).

ID	irMG types	MGFA classification	Combined with thymoma	Antibody profile
1	Pre-existing MG	IV	Yes	AChR-Ab positive
2	Pre-existing MG	V	Yes	AChR-Ab positive
3	Pre-existing MG	IV	Yes	AChR-Ab, anti-titin antibody, anti-RyR antibody positive
4	Pre-existing MG	IV	No	AChR-Ab positive
5	Pre-existing MG	V	Yes	AChR-Ab positive
6	Newly developed MG	V	Yes	AChR-Ab positive
7	Newly developed MG	IV	No	AChR-Ab positive
8	Newly developed MG	V	No	AChR-Ab, anti-titin antibody positive
9	Newly developed MG	III	No	AChR-Ab positive
10	Newly developed MG	V	Yes	AChR-Ab, anti-titin antibody positive
11	Newly developed MG	IV	Yes	AChR-Ab positive
12	Newly developed MG	IV	No	AChR-Ab, anti-titin antibody positive
13	Newly developed MG	IV	Yes	AChR-Ab positive
14	Newly developed MG	V	No	AChR-Ab positive

MG, myasthenia gravis; irMG, immune-related MG; AchR-Ab, acetylcholine receptor antibody; anti-RyR antibody, anti-Ryanodine receptor antibody; MGFA, Myasthenia Gravis Foundation of America.

The median time from exposure to ICI to symptom onset was 15 (6-60) days. 9 cases (39.1%) were classified as grade 3 irAEs, 13 cases (56.5%) as grade 4, and 1 case (4.3%) as grade 5. The most common symptom was eye muscle involvement (n=20, 87%), followed by weakness in the neck and/or limbs (n=15, 65.2%), dyspnea (n=11, 47.8%), and dysarthria and dysphagia (n=10, 43.5%). Among the patients with dyspnea, 6 cases (26.1%) received invasive mechanical ventilation via tracheotomy or tracheal intubation, 5 cases (21.7%) received non-invasive mechanical ventilation.

Laboratory examinations showed that 14 patients were positive for anti-acetylcholine receptor antibody (AChR-Ab), all of whom were irMG patients. All patients were negative for anti-muscle-specific kinase antibody (MuSK-Ab) and anti-low-density lipoprotein receptor-related protein 4 antibody (LRP4-Ab), anti-titin antibody was positive in 4 cases and anti-Ryanodine receptor antibody (anti-RyR antibody) was positive in 1 case. Anti-nuclear antibodies were positive in 11 cases. The median peak value of creatine kinase was 2040 (540 - 26285) U/L. EMG was performed in 17 patients, all of which indicated myogenic damage. 9 patients showed high signal intensity in the quadriceps femoris on T2WI-FS in muscle MRI. 6 patients underwent muscle biopsy, and all biopsies indicated myopathic damage. The auxiliary examination results of patients with myocarditis are shown in **Supplementary Table 1**.

All patients stopped using ICI. 19 patients received methylprednisolone treatment (82.6%), 10 patients received immunosuppressant treatment (43.5%), 11 patients received human immunoglobulin treatment (47.8%), 4 patients received chemotherapy (17.4%), 4 patients received plasma exchange treatment (17.4%), 2 patients received Efgartigimod treatment (8.7%), and 4 patients received temporary cardiac pacemaker implantation due to grade III atrioventricular block (17.4%). The prognosis of irAEs after treatment: complete remission in 13 cases (56.5%), improvement in 7 cases (30.4%), no change in 2 cases (8.7%), and death in 1 case (4.3%), with the cause of death being multiple organ failure.

### 3.2 HLA typing

Among the 23 blood samples of patients, a total of 14 *HLA-A*, 25 *HLA-B*, 16 *HLA-C*, 18 *HLA-DRB1*, 14 *HLA-DQB1*, 11 *HLA-DPB1*, 3 *HLA-DPA1*, 12 *HLA-DQA1*, 3 *HLA-DRB3*, 1 *HLA-DRB4*, 3 *HLA-DRB5* and 4 *HLA-G* alleles were detected. HLA allele frequencies in patients with irMG and myositis, myocarditis are shown in **Table 4**, HLA allele frequencies in patients with myositis and myocarditis are shown in **Table 5**.

**Table 4:** HLA allele frequencies in patients with irMG and myositis, myocarditis (n = 14).

HLA	Allele	2n	%	Control group	HLA	Allele	2n	%	Control group
A	24:02	7	25.0%	15.5%	DQB1	03:03	10	35.7%	15.9%
	02:07	5	17.9%	8.4%		03:01	4	14.3%	21.1%
	02:06	4	14.3%	5.2%		04:01	2	7.1%	4.5%
	11:01	3	10.7%	20.9%		06:01	2	7.1%	10.3%
	02:01	2	7.1%	12.0%		06:02	2	7.1%	7.6%
	Others	7	25.0%			Others	8	28.6%	
B	46:01	6	21.4%	10.3%	DPB1	05:01	10	35.7%	37.0%
	40:01	3	10.7%	9.6%		02:01	6	21.4%	17.6%
	54:01	3	10.7%	3.1%		02:02	2	7.1%	7.7%
	15:01	2	7.1%	4.8%		Others	10	35.7%	
	Others	14	50.0%		DPA1	02:02	16	57.1%	52.2%
C	01:02	10	35.7%	15.9%		01:03	7	25.0%	33.0%
	08:01	3	10.7%	8.5%		02:01	5	17.9%	10.5%
	03:03	2	7.1%	6.9%	DQA1	03:02	8	28.6%	15.2%
	06:02	2	7.1%	8.9%		01:02	4	14.3%	15.2%
	07:02	2	7.1%	15.2%		01:03	3	10.7%	7.6%
	15:02	2	7.1%	3.3%		02:01	3	10.7%	7.2%
	Others	7	25.0%			03:03	2	7.1%	5.0%
DRB1	09:01	8	28.6%	14.7%		05:05	2	7.1%	8.0%
	04:05	3	10.7%	4.8%		06:01	2	7.1%	8.0%
	11:01	3	10.7%	5.6%		Others	4	14.3%	
	07:01	2	7.1%	9.6%	DRB3	02:02	5	17.9%	26.0%
	08:03	2	7.1%	6.3%		03:01	3	10.7%	10.2%
	12:02	2	7.1%	8.7%		Others	1	3.6%	
	Others	8	28.6%		DRB4	01:03	13	46.4%	36.5%
G	01:01	18	64.3%	70.7%	DRB5	01:01	2	7.1%	15.4%
	01:04	9	32.1%	18.5%		Others	1	3.6%	
	Others	1	3.6%						

**Table 5:** HLA allele frequencies in patients with myositis and myocarditis (n = 9).

HLA	Allele	2n	%	Control group	HLA	Allele	2n	%	Control group
A	02:01	5	27.8%	12.0%	DQB1	02:02	3	16.7%	7.6%
	02:07	2	11.1%	8.4%		03:03	3	16.7%	15.9%
	30:01	2	11.1%	5.9%		06:01	3	16.7%	10.3%
	33:03	2	11.1%	8.2%		03:01	2	11.1%	21.1%
	Others	7	38.9%			05:02	2	11.1%	7.3%
B	52:01	3	16.7%	3.0%		Others	5	27.8%	
	13:02	2	11.1%	6.3%	DPB1	02:01	4	22.2%	17.6%
	35:01	2	11.1%	2.4%		04:01	3	16.7%	10.0%
	46:01	2	11.1%	10.3%		05:01	6	33.3%	20.5%
	48:01	2	11.1%	2.5%		Others	5	27.8%	
	Others	7	38.9%		DPA1	01:03	7	38.9%	33.0%
C	03:03	3	16.7%	6.9%		02:02	7	38.9%	52.2%
	07:02	3	16.7%	15.2%		02:01	4	22.2%	10.5%
	01:02	2	11.1%	15.9%	DQA1	02:01	3	16.7%	7.2%
	06:02	2	11.1%	8.9%		01:02	3	16.7%	15.2%
	12:02	2	11.1%	3.1%		01:03	3	16.7%	7.6%
	Others	6	33.3%			03:02	3	16.7%	15.2%
DRB1	07:01	4	22.2%	9.6%		Others	6	33.3%	
	15:02	2	11.1%	3.1%	DRB3	02:02	3	16.7%	26.0%
	16:02	2	11.1%	3.1%		Others	2	11.1%	
	Others	10	55.6%		DRB4	01:03	6	33.3%	36.5%
G	01:01	12	66.7%	70.7%	DRB5	01:02	2	11.1%	6.1%
	01:04	3	16.7%	18.5%		02:02	2	11.1%	5.7%
	Others	3	16.7%			Others	1	5.6%	

The frequencies of the above 12 gene loci were statistically compared with the control group, namely the common and confirmed HLA allele table of the Chinese population (CWD2.4) published by the China Marrow Donor Program Management Center and the large sample HLA literature reports of the Han Chinese population in China (22–27). 14 patients with irMG complicated with myositis and myocarditis, the frequencies of HLA-DQB1*03:03 (35.7% *vs*. 15.9%) and HLA-C*01:02 (35.7% *vs*. 15.9%) were significantly higher than those in the normal population (*P* = 0.016, OR = 2.994, 95% CI = 1.378–6.506). In 9 patients with myositis complicated with myocarditis, the frequency of HLA-B*52:01 locus (16.7% *vs*. 3%) was higher than that in the normal population, and the difference was statistically significant (*P* = 0.018, OR = 6.467, 95% CI = 1.895–23.35). The frequency of HLA-DQB1*03:03 locus in a total of 23 patients was higher than that in the normal population (28.3% *vs*. 15.9%), and the difference was statistically significant (*P* = 0.039, OR = 2.084, 95% CI = 1.056–4.073).

In order to analyze the association between HLA alleles and disease severity, according to the CTCAE classification, 23 patients were divided into two groups: severe group (CTCAE grade 4-5, 14 cases) and moderate group (CTCAE grade 3, 9 cases). We compared the HLA allele frequencies between the two groups, focusing on the three alleles that had previously been statistically different, and the results were as follows: The frequency of HLA DQB1*03:03 in the severe group (32.1%) was higher than that in the moderate group (22.2%), but there was no statistically significant difference (*P* = 0.402). The frequency of HLA-C*01:02 in the severe group (32.1%) was higher than that in the moderate group (16.7%), the difference was not statistically significant (*P* = 0.203). The frequency of HLA-B*52:01 in the moderate group (16.7%) was higher than that in the severe group (0%), and the difference was statistically significant (*P* = 0.032). The association between HLA-B*52:01 and disease severity needs to be further verified. In the future, the sample size will be expanded and a multi-center study will be conducted to further analyze the association between HLA alleles and disease severity.

Because the sample of this study contained a relatively high proportion of thymoma cases, in order to eliminate the bias caused by pathological types on the results, the HLA allele frequencies of thymoma and non-thymoma patients were stratified and compared. Among thymoma patients (n=9), the frequency of *HLA-DQB1 03:03* (33.3% *vs* 15.9%, *P* = 0.057), *HLA-C 01:02* (27.8% *vs* 15.9%, *P* = 0.167), HLA-B*52:01 (5.6% *vs* 3%, *P* = 1.0) was higher than that in the normal population, but the difference was not statistically significant. Among non-thymoma patients (n=14), the frequency of *HLA-DQB1 03:03* (25% *vs* 15.9%, *P* = 0.195), *HLA-C 01:02* (25% *vs* 15.9%, *P* = 0.195), HLA-B*52:01 (7.1% *vs* 3%, *P* = 0.669) was higher than that in the normal population, the difference was also not statistically significant. The frequencies of HLA-DQB1*03:03, HLA-C*01:02 and HLA-B*52:01 in both subgroups were consistently higher than those in the normal population, however, the *P*-value was not significant. This might be caused by the insufficient statistical power after stratification. In this study, the consistent stratified trends but non-significant *P*-values instead suggest that the significant association in the total population is not driven by a single subgroup (such as the thymoma group) alone, but rather the result of the joint contribution of both groups, ruling out the possibility of thymoma as the sole confounding factor.

Sensitivity analysis after excluding thymoma showed that in 14 non-thymoma patients, the associations at the three loci have not reached a significant level, indicating that thymoma is not the sole driving factor for the observed HLA signals. The overall trend (with frequencies higher than those of the normal population) is consistent in both groups, suggesting that the common immune background of the two patient groups may be the main contributor.

## 4 Discussion

Tumor immunotherapy aims to block the activity of inhibitory immune checkpoint proteins and promote T-cell activation, thereby achieving anti-tumor immune effects (28). Due to its safety and accuracy, ICI has a promising future in tumor immunotherapy. Anti-PD-1/PD-L1 inhibitors have become effective ICI and have rapidly become the standard therapy for various cancers (29). Common ligands on macrophages and cancer cells, such as MHC and PDL-1, bind with the receptors TCR and PD-1 on T cells respectively, while TCR and PD-1 typically regulate immune responses. When the immune homeostasis between the positive (TCR/MHC) and negative (CTLA-4/CD80, PD-1/PDL-1) signals for T cell activation is disrupted, cancer cells can evade the host’s immune response and escape from the attack of immune cells, thereby growing and proliferating uncontrollably. In the presence of immune checkpoint inhibitors targeting the CTLA-4/CD80 and PD-1/PDL-1 signaling pathways, these pathways are inhibited, and T cells are reactivated, leading to the death of cancer cells (30).

In addition to restricting the immune response against tumors, CTLA-4 and PD-1 are also important immune checkpoints that help regulate peripheral tolerance to tissue-specific autoantigens. Therapeutic blockade of these checkpoints can disrupt the balance between tolerance and immunity. Clinically this imbalance manifests itself as irAEs, an autoimmune or autoinflammatory toxic reaction associated with checkpoint inhibitors. irAEs can affect almost any tissue, with the main sites of involvement including the skin, the gastrointestinal tract and endocrine organs. In addition, due to the different mechanisms of action of different checkpoint inhibitors, there are differences in their triggering of irAEs. Anti-CTLA-4 drugs exert their effects by enhancing T-cell activation, while PD-1 or PD-L1 blockers are believed to function by reactivating existing CD8+ T-cell responses (31). The overexpression of PD-1 is associated with a favorable prognosis in autoimmune diseases as it contributes to the exhaustion of CD8+ T cells; however, PD-1 inhibitors may exacerbate the symptoms in individuals with MG (32).

The damage caused by immune checkpoint inhibitors in non-tumor sites may be triggered by multiple mechanisms (33), including: increased T-cell clonal expansion leading to the emergence of new self-recognizing effector cells (34), deregulation of inhibition of pre-existing self-recognizing effector T-cells (35), exposure of self-antigens by tumor injury leading to the expansion of self-recognizing clones (36), antibody-mediated autoimmunity (37), a shift towards a more active and less selective innate immune response, direct injury caused by expression of specific molecules on target cells, bystander injury caused by elevated levels of cytokines produced by an active anti-tumor immune response, etc (38).

The potential mechanism of irAEs is believed to be driven by autoimmunity (39), autoimmune diseases are highly polygenic, with many variations in the genome increasing genetic risk. An important feature of the genetics of autoimmune diseases is that variations at the major histocompatibility complex (MHC) locus are closely related to disease risk. Most of these associations are mediated by HLA genes, which play a central role in antigen presentation and immune tolerance (40). The HLA gene, also known as MHC, is located on the short arm of chromosome 6 and can be divided into class II, class III and class I regions. HLA class I genes include HLA-A, B, and C loci, while HLA class II genes include DP, DQ, and DR loci. The MHC proteins encoded by classical HLA genes present self-antigen peptide fragments on the cell membrane for recognition by T cells (41). The HLA complex plays a crucial role in the efficiency and regulation of T-cell-mediated immune responses, with its specific function being to present tumor antigen-derived peptides to the specific T-cell receptors on effector T cells (42), the number of epitopes and the affinity of peptides to HLA molecules are haplotype-specific, so the same antigen may have different immunogenicity in individuals with different HLA genotypes (43). With the development of genome-wide association studies, over 300 susceptibility loci for autoimmune diseases have been identified. Among them, the HLA region plays a significant role in the pathogenesis of most autoimmune diseases, including MG (44). HLA-B*08:01 is a susceptibility gene for early-onset MG (45), *HLA-DR* and *HLA-DQ* alleles predispose patients to late-onset MG and MuSK antibody-positive MG (46). Multiple studies have demonstrated an association between HLA alleles related to autoimmune diseases and the risk of organ-specific irAEs during ICI treatment. There is a statistically significant association between pruritus and HLA-DRB1*11:01, and a nominally significant association between HLA-DQB1*03:01 and colitis (47). HLA-A*26:01, DPA1*01:03 and DPB1*02:01 are associated with thyroid irAEs (48). ICI-related pneumonia is associated with the expression of HLA-B*35 and/or DRB1*11 alleles (43).

Previous reports have indicated that patients with autoimmune diseases may experience a recurrence of their condition during ICI treatment. Our research findings show that 5 patients with a history of MG experienced a recurrence of MG after receiving ICI, confirming that patients with MG have a higher risk of MG relapse when ICI is administered during a stable period of the disease. All 14 patients with irMG combined with myositis and myocarditis in this study had severe conditions, with most of them at MGFA stage IV or V. Among them, 6 patients had myasthenic crisis, and the disease progressed rapidly. The median time from ICI exposure to onset was 15 days. Thymoma is prone to be associated with autoimmune diseases, and the risk of developing autoimmune diseases after the application of ICI is relatively high. In this study, among 9 thymoma patients treated with ICI, 8 developed MG, while the one thymoma patient without MG was of type A. The rest were of type AB-B3. Thymoma of type B has a higher risk of developing irAEs after the application of ICI compared to type A.

Thymoma biology can amplify the HLA-associated risk of ICI–induced adverse events through two synergistic mechanisms. First, thymomas–particularly WHO type B lesions–abnormally express muscle-derived autoantigens such as the acetylcholine-receptor α-subunit, ryanodine-receptor 1, and titin, providing high-affinity peptide substrates for the risk alleles HLA-DQB1*03:03 (class II), HLA-C*01:02 and HLA-B*52:01 (class I) (49). HLA-DQB1*03:03 efficiently presents these peptides to CD4^+^ T cells, while HLA-C*01:02 and HLA-B*52:01 facilitate presentation to CD8^+^ T cells, thereby priming autoreactive T-cell responses (50, 51). Second, thymomas impair central tolerance by reducing AIRE expression and disrupting thymic epithelial cell function, allowing autoreactive T cells that recognize HLA-autoantigen complexes to escape negative selection (52). Upon PD-1/PD-L1 blockade, these escaped T cells are re-activated and readily respond to the HLA-presented autoantigens, intensifying autoimmune pathology (53). Consequently, patients harboring HLA-DQB1*03:03, HLA-C*01:02, or HLA-B*52:01 exhibit a heightened susceptibility to irAEs, an effect that is especially pronounced in the presence of type B thymoma but remains evident even in non-thymoma cohorts, underscoring the independent and synergistic contributions of both thymoma biology and HLA genotype.

Given that class I and class II HLA haplotypes play a crucial role in regulating T-cell immune activation and CTL-mediated tumor cell killing, their differences may play a significant role in determining the therapeutic efficacy and/or irAEs in patients receiving PD-1/PDL-1 blocking monoclonal antibody treatment (54). Due to the rarity of irMG combined with myositis and myocarditis, there have been few studies reporting its HLA typing before, in this study, we conducted HLA typing tests on blood samples from 23 cases of irMG and/or myositis myocarditis, with the aim of identifying high-risk populations at an early stage, the research results show that the frequency of HLA-DQB1*03:03 and HLA-C*01:02 loci in patients with irMG combined with myositis and myocarditis is higher than that in the normal population, and the frequency of HLA-B*52:01 locus in patients with ICI-related myositis and myocarditis is higher than that in the normal population. A study involving 205 MG patients in southern China revealed that DQB1*03:03:02 is positively correlated with the onset of MG in childhood (55), DQB1*03:03 has been confirmed by multiple studies as a genetic risk factor in Japanese MG patients (56, 57), another study about Chinese juvenile- and adult-onset MG patients have found that HLA-C*01:02*:01* is an associated gene for MG (58). Therefore, we speculate that patients carrying the MG-susceptible gene have a higher risk of developing irMG when treated with ICI. Studies have found that MHC class I-deficient non-obese diabetic mice carrying human *HLA-DQ8* develop severe myocarditis and myositis after treatment with anti-PD-1 immune checkpoint inhibitors for cancer (59), however, HLA-B*52:01 has not been reported in myocarditis and myositis. The association of *HLA-DQB1 03:03*, *HLA-C 01:02* with irMG in the present study may reflect both the “underlying susceptibility to MG” and the “amplification effect of ICI-induced immune activation” - patients carrying these alleles have a higher risk of relapse after immunosuppression by ICI. It is more likely to develop autoimmune reactions against the neuromuscular junction. The association of HLA-B*52:01 with myositis and myocarditis has not been seen in previous studies of autoimmune diseases, and may be more inclined towards ICI-specific risk signals.

Our findings suggest that specific HLA alleles may be associated with the risk of ICI-related MG/myositis/myocarditis. The variations and genomic loci identified through this method may highlight those genes and immune pathways that alter the risk of irAEs. These genetic “hit points” can serve as the basis for research to determine the mechanism of irAEs occurrence and may also offer new insights into the targeted killing mechanism required by tumor cells (60).

We acknowledge that our reported mortality (4.3%) is lower than some published series. This may be due to: Early recognition and aggressive management in a specialized MG center. High use of combined immunomodulatory therapies (e.g., steroids, IVIG, plasma exchange, efgartigimod).

Our research has some limitations. This study is a small-sample exploratory analysis of a rare disease, due to the low incidence of irMG and/or myocarditis and myositis, more research is still needed to verify these findings. The identified HLA alleles may be linked to both cancer susceptibility and irAEs risk, and that comparison to an ICI-treated control group is needed for definitive conclusions. The correlation between the response to ICI and irAEs as well as HLA characteristics awaits further exploration in large-scale prospective studies.

## 5 Conclusions

Our research findings reveal that patients carrying autoimmune susceptibility genes have a higher risk of developing irAEs when treated with ICI, specific HLA alleles may be associated with the risk of ICI-related MG/myositis/myocarditis, the abundance of certain HLA alleles or haplotype regions is increased compared with that in the general population. This study is a small-sample exploratory analysis. The association between HLA alleles and irAEs needs to be verified by a large-sample prospective cohort.

## Data Availability

The original contributions presented in the study are included in the article/**Supplementary Material**. Further inquiries can be directed to the corresponding author.
