# Supplementary material for: Clinical features and HLA typing of immune checkpoint inhibitor-associated myasthenia gravis, myocarditis and myositis
Source: Front Oncol. 2025 Nov 21;15:1646231. doi: 10.3389/fonc.2025.1646231 (PMC12678095; doi:10.3389/fonc.2025.1646231)
Supplement: Supplementary file 1 [file Table1.docx]

**Supplementary Table 1.** Auxiliary Examination Results of Patients with ICI-Associated Myocarditis (n = 23)

| **ID** | **Troponin I**  **(ng/mL)** | **CMR** | | | **Coronary artery CT** | **ECG** | **UCG** | | **adjudication level** |
| --- | --- | --- | --- | --- | --- | --- | --- | --- | --- |
|  |  | **Native T1**  **(ms)** | **Native T2**  **(ms)** | **LGE** |  |  | **LVEF, %** | **Echocardiographic Findings** |  |
| 1 | 2.603 | 1120 | 56 | 5.8% of LV myocardial mass (mid-wall, inferior wall) | No stenosis (0%) in all vessels | T-wave inversion in leads Ⅱ, Ⅲ, and aVF | 48 | Left ventricular systolic dysfunction; mild mitral regurgitation | definite |
| 2 | 0.136 | 1085 | 53 | 6.3% of LV myocardial mass (subepicardial, anterior wall) | No stenosis in LM/LAD/LCX; mild stenosis (10%-15%) in RCA proximal segment | ST-segment depression in leads V5-V6; first-degree atrioventricular block | 52 | Mild mitral regurgitation; mild tricuspid regurgitation | definite |
| 3 | 21.993 | NA | NA | NA | No stenosis in LAD/LCX/RCA; mild stenosis (<10%) in LM ostium | Third-degree atrioventricular block | 46 | Left ventricular systolic dysfunction | Probable |
| 4 | 0.58 | 1152 | 59 | 4.9% of LV myocardial mass (linear mid-wall, lateral wall) | No stenosis (0%) in all vessels | ST-segment elevation in leads Ⅲ and aVF | 45 | Left ventricular systolic dysfunction; mild mitral regurgitation; mild tricuspid regurgitation | definite |
| 5 | 0.61 | 1103 | 55 | 7.1% of biventricular myocardial mass (LV inferior wall + RV free wall) | Mild stenosis (15%-20%) in LAD proximal segment; no stenosis in others | T-wave inversion in leads V1-V3 | 48 | Left ventricular systolic dysfunction; mild aortic regurgitation | definite |
| 6 | 0.99 | NA | NA | NA | No stenosis (0%) in all vessels | ST-segment depression in lead aVL; third-degree atrioventricular block | 47 | Left ventricular systolic dysfunction; mild tricuspid regurgitation | Probable |
| 7 | 0.521 | 1092 | 54 | 5.3% of LV myocardial mass (patchy, septum) | Mild stenosis (<10%) in RCA mid-segment; no stenosis in others | Diffuse ST-segment elevation in leads V2-V6 | 45 | Left ventricular systolic dysfunction; mild tricuspid regurgitation | definite |
| 8 | 2.102 | 1135 | 57 | 6.5% of LV myocardial mass (strip-like subepicardial, anterior septum) | No stenosis (0%) in all vessels | T-wave inversion in leads Ⅰ and aVL | 50 | Mild mitral regurgitation | definite |
| 9 | 0.236 | 1168 | 61 | 5.6% of LV myocardial mass (transmural, apex) | Mild stenosis (10%-15%) in LAD diagonal branch; no stenosis in main vessels | ST-segment elevation in lead V3 | 43 | Left ventricular systolic dysfunction; moderate mitral regurgitation; mild tricuspid regurgitation | definite |
| 10 | 0.805 | 1076 | 52 | 6.8% of biventricular myocardial mass (LV septum + RV free wall) | No stenosis (0%) in all vessels | T-wave flattening in leads Ⅱ, Ⅲ, and aVF | 49 | Left ventricular systolic dysfunction | definite |
| 11 | 0.922 | NA | NA | NA | Mild stenosis (<10%) in LCX obtuse marginal branch; no stenosis in main vessels | ST-segment depression in leads V4-V5; first-degree atrioventricular block | 51 | Mild tricuspid regurgitation; trace aortic regurgitation | Probable |
| 12 | 0.486 | 1115 | 56 | 6.2% of biventricular myocardial mass (LV inferior wall + RV anterior wall) | No stenosis (0%) in all vessels | T-wave inversion in leads V5-V6 | 46 | Left ventricular systolic dysfunction; mild mitral regurgitation; trace tricuspid regurgitation | definite |
| 13 | 2.455 | NA | NA | NA | Mild stenosis (15%-20%) in RCA distal segment; no stenosis in main vessels | Ventricular premature beats; complete right bundle branch block | 40 | Left ventricular systolic dysfunction; moderate mitral regurgitation; small pericardial effusion | Probable |
| 14 | 13.815 | 1142 | 58 | 5.7% of biventricular myocardial mass (RV free wall + LV inferior basal segment) | No stenosis (0%) in all vessels | ST-segment elevation in leads Ⅱ, Ⅲ, and aVF | 48 | Left ventricular systolic dysfunction; trace mitral regurgitation; mild tricuspid regurgitation | definite |
| 15 | 0.516 | 1089 | 53 | 4.8% of LV myocardial mass (transmural, apex) | Mild stenosis (<10%) in LAD proximal segment; no stenosis in others | T-wave flattening in leads V1-V2 | 53 | Mild mitral regurgitation; trace tricuspid regurgitation | definite |
| 16 | 0.378 | 1108 | 55 | 5.9% of LV myocardial mass (mid-wall, lateral wall) | No stenosis (0%) in all vessels | ST-segment depression in lead Ⅲ | 54 | Trace mitral regurgitation; mild aortic regurgitation | definite |
| 17 | 0.122 | 1096 | 54 | 5.1% of LV myocardial mass (subepicardial, posterior wall) | Mild stenosis (10%-15%) in LCX mid-segment; no stenosis in others | T-wave inversion in leads V3-V4 | 51 | Mild mitral regurgitation; trace tricuspid regurgitation | definite |
| 18 | 0.128 | 1128 | 57 | 4.6% of LV myocardial mass (punctate, anterior wall) | No stenosis (0%) in all vessels | ST-segment elevation in lead aVF | 50 | Moderate aortic regurgitation; trace mitral regurgitation | definite |
| 19 | 1.35 | NA | NA | NA | Mild stenosis (<10%) in RCA proximal segment; no stenosis in others | ST-segment depression in leads Ⅰ and aVL; third-degree atrioventricular block | 49 | mild tricuspid regurgitation; moderate mitral regurgitation | Probable |
| 20 | 13.284 | NA | NA | NA | No stenosis (0%) in all vessels | Third-degree atrioventricular block | 47 | Left ventricular systolic dysfunction; trace aortic regurgitation; mild mitral regurgitation | Probable |
| 21 | 0.619 | 1112 | 56 | 6.4% of LV myocardial mass (strip-like subepicardial, lateral wall) | Mild stenosis (15%-20%) in LAD diagonal branch; no stenosis in main vessels | T-wave flattening in leads V4-V5 | 55 | Trace mitral regurgitation; mild tricuspid regurgitation | definite |
| 22 | 0.528 | NA | NA | NA | No stenosis (0%) in all vessels | ST-segment elevation in leads V2-V3 | 56 | Mild aortic regurgitation; trace tricuspid regurgitation | Probable |
| 23 | 0.336 | 1098 | 54 | 5.4% of LV myocardial mass (mid-wall, septum + anterior wall) | Mild stenosis (<10%) in LCX obtuse marginal branch; no stenosis in others | ST-segment elevation in leads V2-V4 | 49 | moderate tricuspid regurgitation; trace mitral regurgitation | definite |

Some patients did not undergo CMR due to unstable clinical conditions, mark as Not Available (NA).

Abbreviations: CMR, Cardiac Magnetic Resonance

LV, left ventricle RV, right ventricular

LGE, Late Gadolinium Enhancement

CT, Computed Tomography

ECG, Electrocardiogram,

UCG, Ultrasonocardiography (Echocardiography)

LVEF, Left Ventricular Ejection Fraction,

LM, Left Main Coronary Artery

LAD, Left Anterior Descending Coronary Artery

LCX, Left Circumflex Coronary Artery

RCA, Right Coronary Artery
